# Silent Thyroid, Loud Lipids: The Hidden Link in Asymptomatic Adults

**DOI:** 10.14789/ejmj.JMJ25-0047-OA

**Published:** 2026-01-23

**Authors:** TAHA SUHAIB, SEENA SUGATHAN, NASIR SIDDIQUE, OLUWARONKE JOAN CHIELOTAM OLAKUNORI-OVAGA, IMAD SIBHAI, MOE HEIN, GYANENDRA KC, ADEDAMOLA AGBOOLA, RAYAN TAREG MOHAMED AHMED, LEENA FEISAL MOHAMED ABDALLA, ANKITA SUNIL, IBTISSAM WAEL SAAD, MOHAMED ELMAHI

**Affiliations:** 1 Department of Medicine, Ibne Sina University, Muhammad Medical College, Mirpurkhas, Pakistan; 1 Department of Medicine, Ibne Sina University, Muhammad Medical College, Mirpurkhas, Pakistan; 2 Department of Acute Medicine, Manchester University NHS Foundation Trust, Manchester, United Kingdom; 2 Department of Acute Medicine, Manchester University NHS Foundation Trust, Manchester, United Kingdom; 3 Department of Internal Medicine, Manchester Royal Infirmary Hospital, Manchester, United Kingdom; 3 Department of Internal Medicine, Manchester Royal Infirmary Hospital, Manchester, United Kingdom; 4 Department of Medicine, University of Sunderland, England, United Kingdom; 4 Department of Medicine, University of Sunderland, England, United Kingdom; 5 Department of Medicine, Smt. NHL Municipal Medical College, Ahmedabad, India; 5 Department of Medicine, Smt. NHL Municipal Medical College, Ahmedabad, India; 6 Department of Internal Medicine, Hull University Teaching Hospital, Kingston upon Hull, United Kingdom; 6 Department of Internal Medicine, Hull University Teaching Hospital, Kingston upon Hull, United Kingdom; 7 Department of Radiology, KIST Medical College & Teaching Hospital, Imadol, Lalitpur, Nepal; 7 Department of Radiology, KIST Medical College & Teaching Hospital, Imadol, Lalitpur, Nepal; 8 Department of General Practice, Mersey and West Lancashire NHS Trust, Prescot, United Kingdom; 8 Department of General Practice, Mersey and West Lancashire NHS Trust, Prescot, United Kingdom; 9 Department of Internal Medicine, Sheikh Khalifa Medical City, Abu Dhabi, United Arab Emirates; 9 Department of Internal Medicine, Sheikh Khalifa Medical City, Abu Dhabi, United Arab Emirates; 10 Department of Medicine, Thumbay University Hospital, Ajman, United Arab Emirates; 10 Department of Medicine, Thumbay University Hospital, Ajman, United Arab Emirates; 11 Department of Medicine, American Hospital Dubai, Dubai, United Arab Emirates; 11 Department of Medicine, American Hospital Dubai, Dubai, United Arab Emirates

**Keywords:** subclinical thyroid dysfunction, cardiovascular risk, Billewicz Index, INTERHEART Risk Score, asymptomatic adults

## Abstract

**Background:**

Subclinical thyroid dysfunction is usually asymptomatic, but it potentially disrupts lipid metabolism and increases the risk of cardiovascular disease. We used standardised questionnaires to measure clinical parameters, including subclinical thyroid dysfunction and its relationship with cardiovascular risk in asymptomatic adults.

**Methods:**

A cross-sectional survey was conducted on 501 asymptomatic individuals attending the outpatient clinics from March to August 2025. Data were measured with the Billewicz Clinical Scoring Index (BCSI) for symptoms related to thyroid disease, and the INTERHEART Modifiable Risk Score (IHMRS) was derived to estimate cardiovascular disease risk. Statistical analysis was carried out with the SPSS version 26 (Spearman correlation, Mann-Whitney U and Kruskal-Wallis tests, multivariate linear regression).

**Results:**

There was a moderate positive correlation between BCSI and IHMRS (ρ = 0.382, p < 0.001), with higher mean thyroid-related symptom scores associated with increased cardiovascular risk. Males have significantly higher BCSI scores, and females have substantially greater IHMRS values (p < 0.001). Both scores were associated with older age and higher BMI (p < 0.001). Regression analysis revealed that BCSI, age, female sex, BMI, comorbidities, and medication use were significant positive predictors of IHMRS, while physical activity was inversely associated (p = 0.004).

**Conclusions:**

Subclinical thyroid dysfunction is significantly associated with higher CV risk in asymptomatic adults. Older age, being female, obesity and low physical activity strengthen this relationship. Primary prevention and screening at an earlier age, followed by targeted lifestyle intervention, are essential to avoid future cardiometabolic sequelae.

## Introduction

Subclinical thyroid disease (SCTD), marked by altered levels of TSH and normal levels of free T4 and T3, has attracted clinical interest because it may lead to cardiovascular and metabolic impacts^1)^. Thyroid dysfunction is common in older people, and subclinical forms are found in more than 10% of elderly individuals aged older than 80 years^2)^.

The American Thyroid Association endorses the use of serum TSH levels as a screening test to detect thyroid dysfunction in individuals aged 35 and above, with follow-ups at five-year intervals^3)^. Developments in thyroid diagnostics and the use of TSH and FT4 in specific cases have made it possible to detect subclinical thyroid disorders^4)^.

Thyroid hormones are crucial in lipid metabolism by improving the use, mobilisation, and breakdown of lipids. Hyperthyroidism, whether overt or subclinical, can result in dyslipidemia and minor hyperlipidemia, which can be prevented with thyroid hormone replacement therapy^5, 6)^. Although linked to obesity and other associated metabolic conditions, lipids play crucial physiological roles, such as energy generation, hormone synthesis, and cellular membrane support^7)^.

Thyroid malfunction, especially hypothyroidism, disturbs lipid metabolism, which results in hypercholesterolemia and high cardiovascular risk. Diminished LDL receptor functions and poor control of cholesterol synthesis by thyroid hormones contribute to it^8)^. The complexity of this interaction is further supported by experimental studies indicating that thyroid hormone receptor isoforms have tissue-specific effects on lipid metabolism^9)^.

### Rationale

Thyroid diseases tend to develop silently, and many people are not even diagnosed until they start to get complicated. Among them, lipid metabolism disruptions are another significant and least recognised side effect of mild thyroid disease. Abnormal lipid profiles of asymptomatic adults could be the result of lifestyle or genetic issues, although there could be an underlying thyroid imbalance. This latent connection should be defined because detecting subclinical thyroid disease early is essential to preventing it in time. This approach will enhance lipid regulation and may lower the risk of cardiovascular complications in the long run. The proposed research aims to examine the relationship between thyroid activity and lipid disturbances in symptom-free adult patients, with a particular focus on the importance of preventive screening in the absence of apparent symptoms.

## Materials and Methods

### Primary objective

• To establish the relationship between lipid profile abnormalities and subclinical thyroid dysfunction in asymptomatic adults.

Secondary Objectives

• To examine the occurrence of subclinical thyroid dysfunction in asymptomatic adults.

• To explore the demographic factors (age, gender) of patients with subclinical thyroid dysfunction.

### Methods and ethical considerations

In the current research, a cross-sectional study was used to examine the relationship between subclinical thyroid dysfunction and lipid abnormalities in asymptomatic adults. Patients were recruited from the outpatient clinics of the participating hospitals. The data were collected between March and August 2025 using a structured questionnaire- based tool to gather information on demographics, medical history, and self-reported thyroid and lipid- related symptoms. Eligible participants were approached by trained research assistants who explained the purpose of the research and addressed any questions. Written informed consent was obtained from all participants before data collection. The participants were guaranteed confidentiality, anonymity, and the freedom to withdraw at any time without reprisals.

All the procedures were carried out in accordance with the ethical standards of human research. The ethics review committee of the University of Sunderland, Sunderland, UK (UoS-REC/2025/1001), was consulted and approved the study before data collection began. The integrity and reliability of the research process were ensured by adhering to ethical guidelines and considering the autonomy, privacy, and welfare of participants.

### Recruitment and sampling

The research presupposed the prevalence (p) of 0.5 and an unlimited population, as there was no previous local data. The sample size required was estimated using a 95% confidence interval (Z = 1.96) and a margin of error of 0.05^10)^. Convenience sampling was used to select patients from the outpatient departments of participating hospitals. Of the 540 adults approached, 39 refused to participate or did not meet the eligibility criteria. The other 501 participants, who gave informed consent to the research, filled in the questionnaire and were studied in the final analysis. Convenience sampling was identified as an effective and economical method for recruiting participants, utilising the available time. Nevertheless, it could have had a selection bias, thus restricting the application of the findings to the general population.

### Inclusion and exclusion criteria

The participants were adults aged 18 years and above who attended outpatient departments of the participating hospitals. They had no known thyroid disorder history and were asymptomatic at the time of research. Only individuals who had given informed consent and completed the questionnaire were included. The participants had to be excluded because they either had a diagnosed thyroid condition in the past or had taken thyroid or lipid-lowering pills. The study also excluded pregnant or lactating women, people with chronic systemic conditions (diabetes mellitus, renal disease, cardiovascular disease, etc.), and those who are unwilling and unable to fill out the questionnaire.

### Instruments

The study employed a structured, questionnaire- based instrument with three major sections: demographic data, the Billewicz Diagnostic Index of hypothyroidism, and the INTERHEART Modifiable Risk Score. The questionnaire was designed to holistically assess the association between subclinical thyroid dysfunction and lipid abnormalities in asymptomatic adults. Every component was delivered in English and did not need cultural or linguistic adjustment.

### Demographic information

In the first part, demographic and basic clinical information such as age, gender, body mass index (BMI), marital status, education level, and occupation were collected. This data was utilised to characterise the study population and clarify the demographic factors that may impact thyroid condition and lipid-related symptoms.

### Billewicz Diagnostic Index for hypothyroidism and INTERHEART Modifiable Risk Score

The second part consisted of the Billewicz Diagnostic Index, initially created by Billewicz and his colleagues in 1969 as a clinical instrument to determine the possibility of hypothyroidism based on characteristic symptoms and signs. The index contains 12 clinical features, including weight gain, lethargy, cold intolerance, constipation, dry skin, puffiness, and slow movements, each with a weighted score based on diagnostic relevance. The overall composite score classifies the individuals into euthyroid, borderline, or hypothyroid. Greater (more negative) scores are more likely to presuppose hypothyroidism. The tool has demonstrated internal consistency and reliability, with a Cronbach's alpha of about 0.79, making it suitable for use in clinical and research settings. It was selected for this study as a non-invasive, symptom-based screening tool appropriate for detecting subclinical thyroid dysfunction in asymptomatic adults^11)^. In the third section, the INTERHEART Modifiable Risk Score (IHMRS) was used. This tool was created by Yusuf et al. in 2004 within the framework of the initial INTERHEART study and was subsequently validated in 2010. The IHMRS is a questionnaire that evaluates an individual's risk of developing cardiovascular disease depending on modifiable lifestyle and metabolic issues, such as smoking status, hypertension, diabetes, body weight, eating habits, exercise, psychological stress and alcohol intake. The total for each item creates a cumulative score that classifies people as having low, moderate, or high cardiovascular risk. The instrument is generally considered easy to use, replicable, and applicable across different populations. The IHMRS was applied with prior permission, and the original versions of the surveys were utilised in this study without the need for cultural or linguistic adjustment^12)^.

### Analysis of data

Data were entered into the IBM SPSS Statistics, version 26 (IBM Corp.; Armonk, NY, USA). Descriptive statistics such as frequencies and percentages were used to describe participant demographics and clinical characteristics. Detrended Q-Q plots were used to test for normality of the main study variables, the Billewicz Clinical Scoring Index (BCSI) and INTERHEART Modifiable Risk Score (IHMRS). Nonparametric tests were used because of slight deviations from normality. The association between BCSI and IHMRS was investigated using Spearman's rank-order correlation. The Mann-Whitney U-test was used to analyse gender differences, and the Kruskal-Wallis H test was applied for group comparisons regarding age and BMI. Predictors of IHMRS were identified using multivariate linear regression. Both unstandardized and standardised coefficients, standard errors, t-values, p-values, as well as 95% confidence intervals were presented. The p < 0.05 was considered statistically significant.

## Results

### Demographic characteristics of participants

Table 1 shows the demographic profile of the 501 participants. Most of the participants were aged 60 years or older (N = 216, 43%) and male (N = 270, 54%). The majority had attended primary school (N = 167, 33%), and homemakers were the largest occupational category (n = 160, 33%). With respect to BMI, 133 (27%) were overweight, 132 (26%) were normal weight, and 121 (24%) were obese. Smoking was described as former (N = 196, 39%) or current (N = 190, 38%). The most common medical problems were hypertension (n = 130, 26%), thyroid disease (n = 127, 25%) and cardiac disease (n = 105, 21%). A positive family history for diabetes and heart disease was reported by 149 participants each (30%). Most were taking antihypertensives (N = 145, 29%) or lipid-lowering medications (N = 123, 25%), and moderate physical activity was the most prevalent (N = 174; 35%).

**Table 1:** Demographic characteristics of participants (N = 501)

Variable	f (N)	%		Variable	f (N)	%
Age				Smoking status		
18-29 years	20	4		Never smoked	115	23
30-39 years	45	9		Former smoker	196	39
40-49 years	90	18		Current smoker	190	38
50-59 years	130	26		Known medical conditions		
60 years and above	216	43		Diabetes mellitus	71	14
Gender				Hypertension	130	26
Male	270	54		Thyroid disease (hypo/hyper)	127	25
Female	231	46		High cholesterol/Dyslipidemia	44	9
Educational level				Heart disease (CAD)	105	21
No formal education	127	25		None of the above	24	5
Primary	167	33		Family history		
Secondary/Matric	103	21		Diabetes mellitus	149	30
Intermediate/Higher secondary	33	7		Thyroid disease	116	23
Graduate	29	6		High cholesterol/Heart disease	149	30
Post-graduate	42	8		None of the above/Unknown	87	17
Occupation				Current regular medications		
Student	126	25		Thyroid medication (e.g., levo-thyroxine)	90	19
Employed	138	27		Lipid-lowering drugs (e.g., statins)	123	25
Homemaker	160	33		Antihypertensive	145	29
Unemployed/Retired	77	15		Diabetes medication	98	20
Body mass index (BMI)				None of the above/Don't know	45	9
Underweight (< 18.5)	81	16		Physical activity level		
Normal (18-5-24.9)	132	26		Sedentary (little or no exercise)	126	25
Overweight (25.0-29.9)	133	27		Light (1-2 times/week)	142	28
Obese (≥ 30.0)	121	24		Moderate (3-4 times/week)	174	35
Don't know/Prefer not to say	34	7		Active (5+ times/week)	59	12

Note. Values represent frequency (n) and percentage (%) of participants (N = 501)

### Normality assessment of study variables

**Figure 1:**
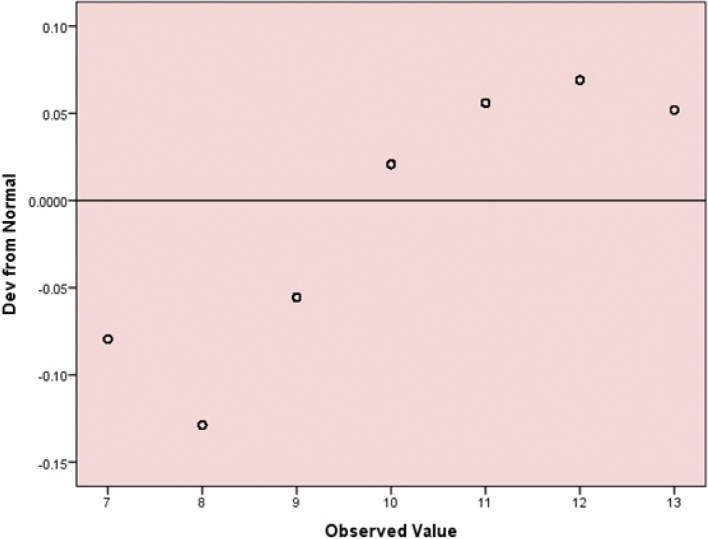
Detrended normal Q-Q plot for total Billewicz Clinical Scoring Index (BCSI) scores (N = 501)

Figure 1 shows a detrended normal Q-Q plot illustrating the deviations of the observed values of the total Billewicz Clinical Scoring Index (BCSI) from the expected normal distribution. The points represent standardised residuals plotted against their expected normal values, with the horizontal line at zero indicating perfect normality. Minor and randomly scattered deviations around the zero line suggest an approximately normal distribution, whereas larger systematic deviations would indicate departures from normality. In this plot, the data points show mild variation from the zero line, suggesting slight but not substantial deviation from normality.

**Figure 2:**
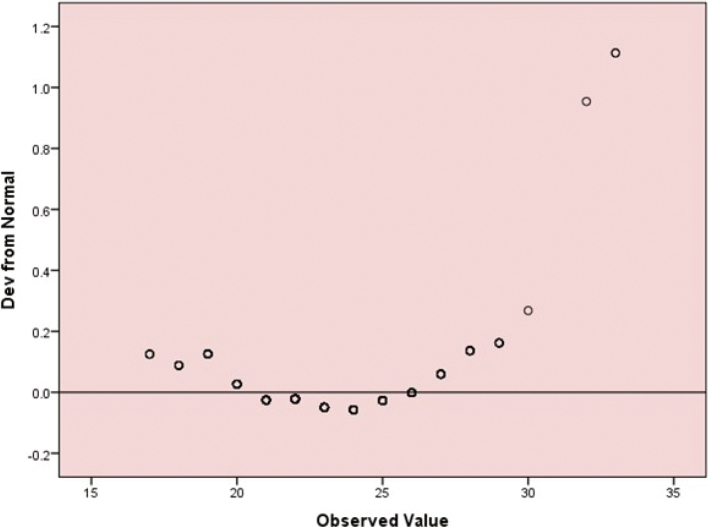
Detrended normal Q-Q plot for total INTERHEART Risk Scores (IHMRS) scores (N = 501)

Figure 2 shows that this detrended normal Q-Q plot displays the deviations of the observed values of the total INTERHEART Modifiable Risk Score (IHMRS) from the expected normal distribution. Each point represents the difference between the observed and expected standardised values. The horizontal line at zero indicates perfect normality. The systematic upward curvature, particularly at higher observed values, suggests positive skewness and departure from normality. This pattern indicates that the distribution of the total IHMRS scores deviates from the normal distribution, with more extreme high values than expected under normality.

### Correlation between billewicz clinical scoring index and INTERHEART Risk Scores

Table 2 shows the Spearman's correlation matrix between the Billewicz Clinical Scoring Index (BCSI) and INTERHEART Risk Scores (IHMRS) for 501 participants. There was a significant positive correlation (ρ = 0.382, t = 9.23, p < 0.001) between the BCSI and cardiovascular risk as assessed by IHMRS. Higher BCSI scores were actually moderately associated with a higher CVD risk.

**Table 2:** Spearman's correlations among study variables (N = 501)

Variables	Spearman's ρ	t(df)	p
Billewicz Clinical Scoring Index (BCSI)	-	-	-
INTERHEART Risk Scores (IHMRS)	0.382	9.23	<0.001**

Note. Values are Spearman's rank-order correlation coefficients (ρ); p < 0.001 (**), N = 501; All percentages used in descriptive analysis are based on valid responses (i.e., excluding missing data) to ensure consistency across variables.

### Gender differences in BCSI and IHMRS

Table 3 shows the gender differences of BCSI and IHMRS. Men scored significantly higher on BCSI (Mean Rank = 278.15) compared with women (Mean Rank = 218.92; U = 26,550, z = -3.823, p < 0.001), while women had significantly higher IHMRS scores (Mean Rank = 292.47) in comparison with men (Mean Rank = 214.83; U = 22,345, z = -4.210, p < 0.001). These findings demonstrate significant gender differences in the presentation and cardiovascular risk profile.

**Table 3:** Mann-Whitney U Tests Comparing Gender Differences in Billewicz Clinical Scoring Index (BCSI) and INTERHEART Risk Scores (IHMRS) (N = 501)

Variable	Gender	N	Mean rank	Sum of ranks	U	Z	p
Billewicz Clinical Scoring Index (BCSI)	Male	270	278.15	75,100.00	26,550.000	-3.823	< 0.001**
	Female	231	218.92	50,570.00			
INTERHEART Risk Scores (IHMRS)	Male	270	214.83	58,004.00	22,345.000	-4.210	< 0.001**
	Female	231	292.47	67,666.00			

Note. N = 501 (Males = 270, 54%; Females = 231, 46%); Mann-Whitney U test was used for all comparisons; p values marked with ** indicate statistical significance at p < 0.001

### Age-related differences in BCSI and IHMRS

Table 4 illustrates the difference in BCSI and IHMRS distribution between different age groups. Both BCSI and IHMRS scores correlated with age, with the least mean ranks at 18-29 years (BCSI = 155.4; IHMRS = 162.55) and highest for those of 60+ years of age (BCSI = 348.9; IHMRS = 342.85). There were significant age differences in BCSI (χ^2^ (4) = 38.41, p < 0.001) and IHMRS (χ^2^ (4) = 31.88, p < 0.001), indicating that older participants had higher clinical and cardiovascular risk scores than the younger subjects.

**Table 4:** Kruskal-Wallis Tests Comparing Total Billewicz Clinical Scoring Index (BCSI), and INTERHEART Risk Scores (IHMRS) Across Age Groups (N = 501)

Variable	Age group	N	Mean rank	x^2^(df = 4)	p
Billewicz Clinical Scoring Index (BCSI)	18-29 years	20	155.4	-	-
	30-39 years	45	198.25	-	-
	40-49 years	90	245.1	-	-
	50-59 years	130	305.3	-	-
	60 years and above	216	348.9	38.41	<0.001**
INTERHEART Risk Scores (IHMRS)	18-29 years	20	162.55	-	-
	30-39 years	45	210.4	-	-
	40-49 years	90	259.75	-	-
	50-59 years	130	310.25	-	-
	60 years and above	216	342.85	31.88	<0.001**

Note. N = number of participants in each age category; % = percentage of the total sample; Percentages are based on total N = 501; Values are mean ranks from Kruskal-Wallis H tests; Overall test statistics are reported in the bottom row for each variable: Total BSCI (χ^2^(4) = 38.41, < 0.001**), and Total IHMRS (χ^2^(4) = 31.88, < 0.001); Significance levels: p < 0.001**

### Body Mass Index (BMI) differences in BCSI and IHMRS

Table 5 presents a comparison of the Billewicz Clinical Scoring Index (BCSI) and INTERHEART Risk Scores (IHMRS) across different BMI grades in 501 patients. Both BCSI and IHMRS scores increased with increasing BMI, with the lowest mean rank for underweight (BCSI = 192.65; IHMRS = 210.57) and the highest for obese subjects (BCSI = 333.42; IHMRS = 339.94). Differences were significant among BMI categories for BCSI (χ^2^(4) = 27.66, p < 0.001) and IHMRS (χ^2^(4) = 34.92, p < 0.001), reflecting that individuals with higher BMI have increasing clinical and cardiovascular risk scores.

**Table 5:** Kruskal-Wallis Tests Comparing Total Billewicz Clinical Scoring Index (BCSI), and INTERHEART Risk Scores (IHMRS) Across Body Mass Index (N = 501)

Variable	Body mass index (BMI) category	N	Mean rank	x^2^(df = 4)	p
Billewicz Clinical Scoring Index (BCSI)	Underweight (< 18.5)	81	192.65	-	-
	Normal (18.5-24.9)	132	225.48	-	-
	Overweight (25.0-29.9)	133	287.76	-	-
	Obese (≥ 30.0)	121	333.42	-	-
	Don't know/Prefer not to say	34	251.35	27.66	< 0.001**
INTERHEART Risk Scores (IHMRS)	Underweight (< 18.5)	81	210.57	-	-
	Normal (18.5-24.9)	132	238.49	-	-
	Overweight (25.0-29.9)	133	296.88	-	-
	Obese (≥ 30.0)	121	339.94	-	-
	Don't know/Prefer not to say	34	254.12	34.92	< 0.001**

Note. N = number of participants in each age category; % = percentage of the total sample; Percentages are based on total N = 501; Values are mean ranks from Kruskal-Wallis H tests; Overall test statistics are reported in the bottom row for each variable: Total BSCI (χ^2^ (4) = 27.66, < 0.001**), and Total IHMRS (χ^2^ (4) = 34.92, < 0.001); Significance levels: p < 0.001**

### Predictors of cardiovascular risk (IHMRS): multiple linear regression analysis

Table 6 shows the multiple linear regression analysis of predictors of the INTERHEART Modifiable Risk Score (IHMRS). The model found multiple variables as significant determinants of cardiovascular risk. The Total Billewicz Clinical Scoring Index (BCSI) was positively correlated with IHMRS (B = 0.318, p < 0.001), suggesting that higher thyroid- related symptom scores are associated with a higher cardiovascular risk. Age (B = 0.262, p = 0.001), female sex (B = 1.086, p = 0.005), BMI (B = 0.298, p = 0.001). Higher body mass index was significantly associated with increased IHM resistance overall. In contrast, greater weekly physical activity was related to lower IHMRS (B = -0.314, p = 0.004). These findings indicate that net individual and population effects of clinical, demographic and lifestyle factors contribute collectively to cardiovascular risk by IHMRS.

**Table 6:** Multiple Linear Regression Predicting INTERHEART Modifiable Risk Score (IHMRS) from Clinical and Demographic Factors (N = 403)

Predictor	B	SE	β	t	p	95% CI LL	95% CI UL
Constant (INTERHEART Modifiable Risk Score)	14.821	1.122	-	13.21	< 0.001***	12.61	17.03
Total Billewicz Clinical Scoring Index (BCSI)	0.318	0.071	0.168	4.48	< 0.001***	0.178	0.458
Age	0.262	0.081	0.133	3.23	0.001**	0.102	0.422
Gender (Female = 1)	1.086	0.381	0.117	2.85	0.005**	0.335	1.837
BMI	0.298	0.089	0.141	3.35	0.001**	0.123	0.473
Medical conditions	0.207	0.072	0.120	2.88	0.004**	0.065	0.349
Current regular Medication	0.241	0.087	0.110	2.77	0.006**	0.070	0.412
Physical activity (Weekly)	-0.314	0.108	-0.122	-2.91	0.004**	-0.526	-0.102

Note. N = 501, B = unstandardized regression coefficient; SE = standard error; β = standardized regression coefficient; CI = confidence interval; LL = lower limit; UL = upper limit; All predictors were entered; p < 0.01**, p < 0.001***.

## Discussion

In the current study, we attempted to evaluate the relationship between subclinical thyroid dysfunction and cardiovascular (CV) risk using a combination of the Billewicz Clinical Scoring Index (BCSI) and the INTERHEART Modifiable Risk Score (IHMRS). We found a moderate positive correlation between the Billewicz Clinical Scoring Index and the INTERHEART Risk Score, indicating that patients with more severe thyroid-related clinical signs also had a higher risk of experiencing CVD. This is consistent with earlier studies that have demonstrated an association between hypothyroidism and adverse cardiometabolic outcomes (such as dyslipidemia, cardiac dysfunction and cardiovascular events) in addition to positive effects of thyroid hormone replacement on these risk factors^13)^.

In the present study, males had higher scores on the Billewicz Clinical Scoring Index (BCSI) than females, indicating a greater severity of thyroid-related clinical signs. This is consistent with earlier studies that showed the prevalence of hypothyroidism symptoms had more predictive power in men than women^14)^. Conversely, females had significantly higher INTERHEART Risk Scores than males, suggesting an adverse cardiovascular risk profile. This result is also similar to the previous research, which found that women had a higher risk of CVD due to various cardiovascular risk factors and were more frequently off target than men^15)^.

In our research, we also found that BCSI significantly increased with age, suggesting that older individuals exhibited more severe thyroid function. This observation is also in agreement with results that demonstrate the expression of thyroid hormone levels to be altered throughout ageing, resulting in more severe clinical presentation in elderly individuals^16)^. Similarly, INTERHEART Risk Scores were significantly higher among older subjects, suggesting the predominance of cardiovascular risk in this group. This is in line with recent evidence demonstrating that age is an influential independent risk factor for CVD, which explains the age-dependent increase in the INTERHEART score^17)^.

In our research, thyroid-related clinical features were more relevant to increasing BMI, suggesting a possible relationship between high body mass and thyroid disorders. This is corroborated by a meta-analysis, which suggested that obesity is significantly associated with the risk of overt and subclinical hypothyroidism and positive thyroid antibodies, indicating that increased BMI might be aggravating thyroid patterns^18)^. INTERHEART Risk Scores also increased with increasing BMI, implying that the overweight and obese have an overall higher cardiovascular risk. This finding is consistent with large-scale studies that have seen causal effects of higher adiposity on the risk of coronary heart disease, hypertension and various cardiovascular outcomes^19)^.

The significant associations of thyroid symptom score, age, female gender and higher BMI with increased INTERHEART Modifiable Scores were also substantiated by our multiple regression analysis. Taken together, these results suggest that metabolic factors and demographics contribute significantly to CVD risk. The associations are in line with previous reports demonstrating that hypothyroidism, older age, female sex and adiposity accentuate the degree of cardiometabolic discordance^13, 15, 17), 19)^.

In our analysis, comorbidity and current medication usage were both independently correlated with high INTERHEART Modifiable Risk Scores, suggesting that participants with comorbid illness or a history of chronic drug use contribute more towards the development of cardiovascular risk. This is consistent with previous research that suggests patients who have comorbid diseases such as cardiovascular or renal disease, particularly those on chronic medicine, have a greater incidence of cardiovascular events^20)^. Finally, in our study, physical activity was negatively associated with INTERHEART Modifiable Risk Scores, indicating the protective role of moderate to vigorous physical activity on cardiovascular risk. This is consistent with earlier research that some degree of total daily physical activity was inversely associated with the incidence of CVD in hypertensive adults^21)^.

Although informative, the study had limitations. The nature of the cross-sectional design prevents us from making causal inferences between subclinical thyroid dysfunction and lipid abnormalities. Speaking of selection, the convenience sampling from the hospital outpatient department likely led to selection bias and decreased generalizability as applied to the general population. Moreover, using clinical scores (BCSI and IHMRS) instead of biochemical tests to evaluate thyroid and lipid levels might have led to under- or overestimation of the actual prevalence of subclinical disease. Reported physical activity and medical history findings might also have been biased due to recall.

### Recommendations

The causal directionality of the relationship between thyroid dysfunction and lipid abnormalities needs to be examined in longitudinal studies or addressed with cohort studies. The addition of biochemical investigations such as serum TSH, FT4, FT3 and lipid profile would improve the diagnostic accuracy. Research should also examine genetic, dietetic, and psychosocial antecedents that could moderate or mediate this association. Further, community research with a more heterogeneous population could enhance the external validity. Intervention trials that evaluate the effects of early thyroid hormone replacement or lifestyle changes on lipid results would bring this finding one step closer to clinical relevance.

## Conclusion

This research emphasises a strong link between subclinical thyroid dysfunction and cardiovascular risk among asymptomatic adults. Higher Billewicz scores were moderately associated with higher INTERHEART risk scores, with the association being modified by age, gender and anthropometric data, i.e. BMI. Results further highlight the covert yet profound contribution of thyroid imbalance to lipid and cardiovascular risk profiles, despite being free of obvious symptoms. Screening at earlier stages of subclinical thyroid disease, especially among older or overweight individuals, could be a key to preventive action from future cardiovascular morbidities. This may imply the need for increased awareness, lifestyle change and subsequent screening of the thyroid gland in asymptomatic individuals as a strategy to help improve long-term cardiovascular health.

## Author contributions

TS conceived and designed the study, supervised data collection, and critically reviewed the manuscript. SS contributed to the study design, interpretation of the Billewicz Clinical Scoring Index (BCSI) outcomes, and drafting of the methodology section. NS assisted with data acquisition and clinical interpretation. OJCO and IS were responsible for participant recruitment and management of outpatient data collection. MH and GKC supported statistical analysis and data visualisation. AA contributed to the interpretation of INTERHEART Modifiable Risk Score (IHMRS) results and revision of the manuscript. RTMA and LFM assisted with the literature review and drafting of the results section. AS and IWS performed data entry and ensured data quality and consistency. ME contributed to the discussion, proofreading, and final revision of the manuscript. All authors read and approved the final version of the manuscript.

## Conflicts of interest statement

The authors declare that there are no conflicts of interest.
